# Deep Reinforcement Learning-Guided Inverse Design of Transparent Heat Mirror Film for Broadband Spectral Selectivity

**DOI:** 10.3390/ma18122677

**Published:** 2025-06-06

**Authors:** Zhi Zeng, Haining Ji, Tianjian Xiao, Peng Long, Bin Liu, Shisong Jin, Yuxin Cao

**Affiliations:** School of Physics and Optoelectronics, Xiangtan University, Xiangtan 411105, China

**Keywords:** transparent heat mirror, spectrally selective properties, deep reinforcement learning, inverse design, building energy efficiency

## Abstract

With the increasing energy consumption of buildings, transparent heat mirror films have been widely used in building windows to enhance energy efficiency owing to their excellent spectrally selective properties. Previous studies have typically focused on spectral selectivity in the visible and near-infrared bands, as well as single-parameter optimization of film materials or thickness, without fully exploring the performance potential of the films. To address the limitations of traditional design methods, this paper proposes a deep reinforcement learning-based approach that employs an adaptive strategy network to optimize the thin-film material system and layer thickness parameters simultaneously. Through inverse design, a Ta_2_O_5_/Ag/Ta_2_O_5_/Ag/Ta_2_O_5_ (42 nm/22 nm/79 nm/22 nm/40 nm) thin-film structure with broadband spectral selectivity was obtained. The film exhibited an average reflectance of 75.5% in the ultraviolet band and 93.2% in the near-infrared band while maintaining an average visible transmittance of 87.0% and a mid- to far-infrared emissivity as low as 1.7%. Additionally, the film maintained excellent optical performance over a wide range of incident angles, making it suitable for use in complex lighting environments. Building energy simulations indicate that the film achieves a maximum energy-saving rate of 17.93% under the hot climatic conditions of Changsha and 16.81% in Guangzhou, demonstrating that the designed transparent heat mirror film provides a viable approach to reducing building energy consumption and holds significant potential for practical applications.

## 1. Introduction

The accelerating urbanization process has given rise to pressing challenges, including critical energy shortages and environmental degradation that have reached unprecedented levels of severity [1,2,3]. Building energy consumption accounts for more than 40% of global energy consumption, and this proportion is expected to continue to increase with the ongoing improvement in living standards [4,5,6]. Windows, the least thermally insulated component of the building envelope, contribute to approximately 60% of the building energy consumption [7,8,9,10,11]. The solar spectrum can be divided into ultraviolet (UV, 0.2–0.38 μm) light, visible (VIS, 0.38–0.78 μm) light, and near-infrared (NIR, 0.78–2.5 μm) light, which account for 3%, 43%, and 54% of the total solar energy, respectively [12,13,14]. Among these, the thermal effect of NIR radiation is the primary contributor to indoor heat gain, while UV radiation not only damages human skin but is also absorbed by materials, reducing their durability [15,16,17]. Therefore, it is essential to design a smart window with high transmittance in the visible band and high reflectance in the UV and NIR bands.

Transparent heat mirror film (THM) has demonstrated significant potential in the development of smart windows due to its high transmittance in the visible spectrum and outstanding reflectance in the infrared band. Early on, Fan et al. [18] pioneered the design of a TiO_2_/Ag/TiO_2_-based THM structure, laying the groundwork for the development of dielectric/metal/dielectric (D/M/D) multilayers. Since then, D/M/D configurations have garnered widespread attention owing to their superior optical properties. Dalapati et al. [19] investigated the TiO_2_/Cu/TiO_2_ structure, achieving an average visible transmittance of up to 90%, along with 85% NIR reflectance. Similarly, Sibin et al. [20] optimized the ITO/Ag/ITO structure by adjusting the thickness of each layer and etching the glass substrate, resulting in an average visible transmittance of 91% while maintaining high NIR reflectance. However, the D/M/D structure still exhibits certain limitations in spectrally selective modulation and relies on a parametric scanning approach based on repeated trial-and-error processes for thin-film design, which is not only inefficient but also cost-prohibitive. To address these challenges, researchers have incorporated intelligent algorithms to optimize multilayer dielectric/metal interlayer structures, further enhancing film performance. For example, Hong et al. [14] investigated the Si_3_N_4_/Ag/Si_3_N_4_/Ag/Si_3_N_4_; structure and optimized the thickness of each layer using a particle swarm optimization algorithm, achieving an average visible transmittance of 88.6% and an average NIR reflectance of 92%. However, previous studies have primarily focused on single-parameter optimization, such as the material choice or thickness, without fully exploring the potential for enhancing the films’ performance. Moreover, traditional intelligent algorithms often struggle with high-dimensional data, making the simultaneous optimization of both the material composition and thickness a significant challenge.

Machine learning is widely applied in material preparation, performance prediction, and structural design, offering new research perspectives [21,22]. It can autonomously extract features from data and outperforms traditional intelligent algorithms in handling large-scale and high-dimensional datasets [23,24,25]. Deep reinforcement learning (DRL) combines the feature extraction capability of deep learning with the decision-making ability of reinforcement learning [26,27,28] and has emerged as one of the most prominent research directions in machine learning [29].

In this paper, a deep reinforcement learning-based approach is employed to simultaneously optimize both the material system and thickness of the thin film using an adaptive strategy network, enabling the inverse design of film structures with broadband spectral selectivity. The optimized results are compared to obtain the film with the best comprehensive performance. The underlying mechanism responsible for spectral selectivity is investigated through an electromagnetic field distribution analysis. Furthermore, by examining the influences of the incident angle and polarization state on the optical properties of the film, it is demonstrated that its optical performance remains stable under varying incident conditions. Finally, building energy consumption simulations under the hot climate of Changsha and Guangzhou, China, further validate the film’s outstanding energy-saving capacity.

## 2. Methodology

### 2.1. Data Preparation and Preprocessing

**Figure 1:**
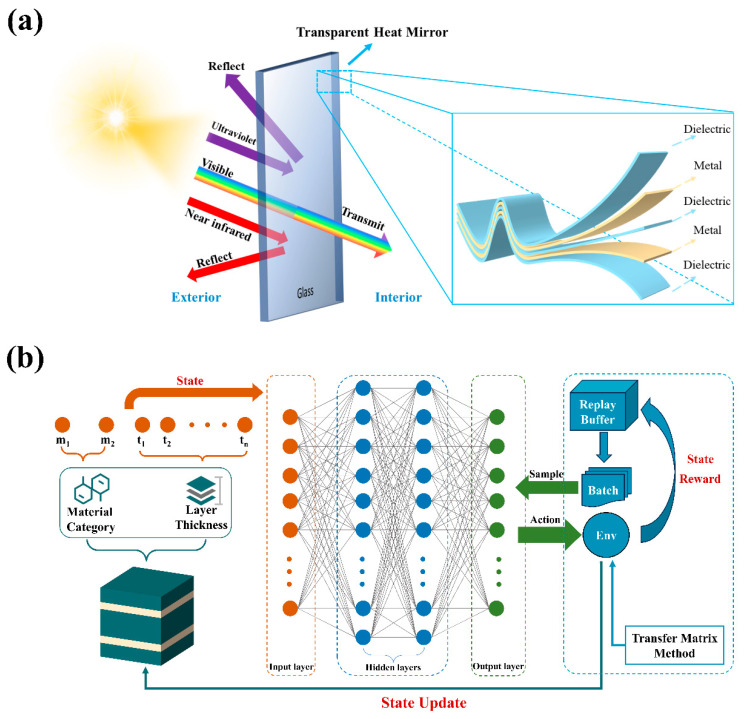
(**a**) Diagram of the working mechanism of a transparent heat mirror film. (**b**) Schematic diagram of the deep Q-network model framework.

An ideal transparent heat mirror film transmits all visible light while completely reflecting UV and NIR radiation, as illustrated in Figure 1a. To achieve this functionality, THM films typically utilize high-reflectivity precious metals such as Au, Ag, and Cu as intermediate metallic layers [30]. In recent years, TiN has also been considered due to its lower cost [31]. Additionally, dielectric materials serve as antireflective layers to minimize reflection losses in the visible spectrum. Commonly used dielectric materials include Al_2_O_3_, AlN, HfO_2_, ITO, Si_3_N_4_, SiO_2_, Ta_2_O_5_, TiO_2_, ZnO, ZnS, WO_3_, and AZO, among others.

In this paper, to facilitate material optimization using machine learning, we assigned integer codes to the selected materials, as shown in Table S1. Since some of the collected data contained missing values, we applied linear interpolation over the wavelength range of 0.28 μm to 2.5 μm using 500 evenly spaced interpolation points. This process standardized the wavelength data across different materials, providing a consistent dataset for subsequent optimization.

### 2.2. Optimization Framework Design

In this study, a deep Q-network (DQN) framework (as shown in Figure 1b) was developed to optimize both the material system and thickness parameters of thin films. The material system, composed of a periodic stack of dielectric and metal layers, is represented by a state defined as $\left\{m_{1},m_{2},t_{1},t_{2},\ldots ,t_{n}\right\}$, where $m_{1},m_{2}$ denote the material codes for dielectric and metal layers, respectively, and $\left\{t_{1},t_{2},\ldots ,t_{n}\right\}$ represent the thicknesses of each layer. The number of layers, *n*, is determined by the specific configuration of the film. The action set is defined as $\left\{m_{1}+1,m_{1}-1,m_{2}+1,m_{2}-1,t_{1}+\Delta t,t_{1}-\Delta t,\ldots ,t_{n}+\Delta t,t_{n}-\Delta t\right\}$, where Δt is fixed at 1 nm. To maximize the sum of ultraviolet reflectance and visible transmittance, and near-infrared reflectance, the reward function is defined as follows:

(1) $$r={\bar{R}}_{\mathrm{UV}}+{\bar{T}}_{\mathrm{VIS}}+{\bar{R}}_{\mathrm{NIR}}$$

where ${\bar{R}}_{\mathrm{UV}}$, ${\bar{T}}_{\mathrm{VIS}}$, and ${\bar{R}}_{\mathrm{NIR}}$ represent the average ultraviolet reflectance, the average visible transmittance, and the average near-infrared reflectance, respectively.

**Figure 2:**
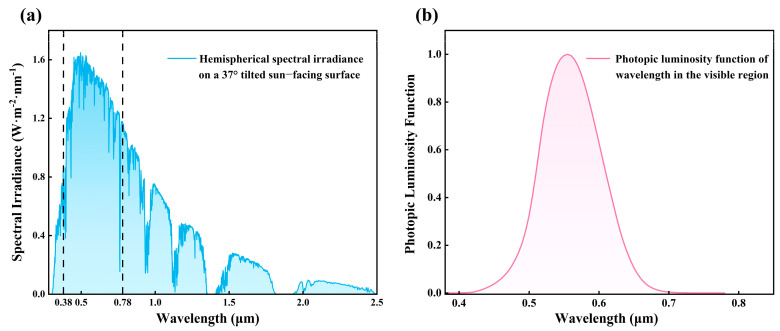
(**a**) Solar spectral irradiance under AM1.5 atmospheric conditions. (**b**) Photopic luminosity function of the human eye in the visible light range [32].

Solar radiation in the ultraviolet (UV) range is typically divided into three regions: UVC (200–280 nm), UVB (280–315 nm), and UVA (315–380 nm). Among these, UVC is almost completely absorbed by the ozone layer, while UVA has the most significant impact on the human body, as it can penetrate the epidermis and reach the dermis [32]. Therefore, in this study, we focus on the UV spectrum within the range of 280–380 nm. The average reflectance and transmittance values, ${\bar{R}}_{\mathrm{UV}}$, ${\bar{T}}_{\mathrm{VIS}}$, and ${\bar{R}}_{\mathrm{NIR}}$, are calculated using the following equations [19,33]:

(2) $${\bar{R}}_{\mathrm{UV}}=\frac{\int_{0.28}^{0.38}R(\lambda )S(\lambda )d\lambda }{\int_{0.28}^{0.38}S(\lambda )d\lambda }$$

(3) $${\bar{T}}_{\mathrm{VIS}}=\frac{\int_{0.38}^{0.78}T(\lambda )V(\lambda )d\lambda }{\int_{0.38}^{0.78}V(\lambda )d\lambda }$$

(4) $${\bar{R}}_{\mathrm{NIR}}=\frac{\int_{0.78}^{2.5}R(\lambda )S(\lambda )d\lambda }{\int_{0.78}^{2.5}S(\lambda )d\lambda }$$

in Equations (2) and (4), R(λ) represents the reflectance of the thin film, and S(λ) denotes the solar spectral irradiance under AM1.5 atmospheric conditions, as illustrated in Figure 2a. In Equation (3), T(λ) represents the transmittance of the thin film, while V(λ) corresponds to the photopic luminosity function of the human eye, as shown in Figure 2b. The function V(λ) is zero outside the visible spectrum and reaches a peak value of 1 at a wavelength of 0.55 μm, indicating the highest sensitivity of the human eye at this wavelength. The transmittance T(λ) and reflectance R(λ) of the film are calculated using the transfer matrix method (TMM), which is one of the most commonly used methods for analyzing the optical properties of multilayer films based on the refractive index (n), extinction coefficient (k), film thickness, and incident light wavelength [34].

According to the law of energy conservation and the law of thermal radiation, the spectral emissivity ε(λ) of the film can be calculated from its reflectance R(λ) and transmittance T(λ) as follows [35,36]:

(5) ε(λ)=A(λ)=1−T(λ)−R(λ)

Therefore, the average emissivity of the film in the mid- to far-infrared range (2.5–25 μm) can be calculated using the following expression [37]:

(6) $$\bar{\varepsilon }=\frac{\int_{2.5}^{25}\varepsilon (\lambda )B(\lambda ,Τ)d\lambda }{\int_{2.5}^{25}B(\lambda ,Τ)d\lambda }$$

where B(λ,Τ) is the spectral radiance of a blackbody at temperature T = 300 K given by Planck’s law [38].

### 2.3. Model Training

The DQN model employs two deep neural networks: the main network and the target network. Both networks share the same architecture, which is a fully connected feedforward neural network comprising an input layer (with $n_{\mathrm{input}}$ nodes representing the encoded thin-film structure), a single hidden layer with 64 neurons activated by the Tanh function, and an output layer with $n_{\mathrm{output}}$ nodes corresponding to the discrete action space (i.e., modifications to the material type or layer thickness). The main network is responsible for computing the Q-values of different actions in the current state, and its weight parameters are continuously updated during training. In contrast, the target network is used to compute the Q-values of all possible actions in the next state. To maintain stability, the weights of the target network are synchronized with those of the main network every 2000 steps. This dual-network design effectively enhances the stability of the model and mitigates the risk of Q-value overestimation, which could otherwise lead to suboptimal policy choices [39].

During the decision-making process, the agent perceives the current state from the environment and uses the main network to compute the Q-values of all possible actions. An action is then selected using an ε-greedy strategy: with probability ε, a random action is chosen (exploration), and with probability 1−ε, the action with the highest Q-value is selected (exploitation). In this study, the ε-greedy strategy is used to dynamically adjust ε so that the model can explore more in the early stage of training and gradually tend to the optimal strategy in the late stage of training to accelerate convergence. This approach helps accelerate convergence toward the optimal policy.

The agent interacts with the environment by performing actions, which in turn update the environment's state and provide corresponding reward feedback. The agent then uses the updated state to compute new Q-values and decides whether to explore or exploit based on the updated ε. Through this continuous loop of interaction and learning, the agent incrementally improves its policy by acquiring new experience from the environment. Unlike supervised learning, DQN does not require pre-existing training data; instead, it learns by interacting with a TMM-based simulation environment that provides performance feedback for each candidate structure.

DQN employs an experience replay mechanism, where at each time step, the original state (s), action (a), reward (r), next state $\left(s^{’}\right)$, and a termination flag (done) are stored in the experience replay buffer. During training, a random batch of experiences is sampled from the buffer and used to update the main network. In this study, we set the experience buffer size to 20,000 and the batch size to 64.

Meanwhile, the target network computes the target Q-values for the next state in the sampled batch. The model uses the mean squared error (MSE) loss function to calculate the loss between the target Q-values and Q-values of the current state. The gradient descent algorithm is then applied to update the weight parameters of the main network.

A sensitivity analysis of the model was conducted by varying key parameters, including the random initialization seed, the number of training episodes, the maximum number of steps per episode, and the learning rate. The results indicated that the model converged reliably only when the number of training episodes reached 20 or more. Therefore, the number of training episodes in this study was set to 20. As training progresses, the Q-value function gradually converges, enabling the agent to select actions that maximize long-term rewards in any given state. Ultimately, the model outputs the state that yields the highest reward, which corresponds to the optimal thin-film structure parameters.

### 2.4. Optical Simulations

The optical simulations of the designed thin-film structure were performed using COMSOL Multiphysics^®^ (v6.2, COMSOL AB, Stockholm, Sweden) [40]. COMSOL is based on the finite element method (FEM), which discretizes the computational domain into numerous fine meshes for numerical analysis. The model was constructed with an edge length of 1 μm, and Floquet periodic boundary conditions were applied to simulate the optical response of an infinite periodic array. By analyzing the electromagnetic field distribution, the internal mechanisms enabling spectral selectivity in the film structure were investigated.

In practical applications, sunlight does not always strike window surfaces perpendicularly. The angle of incidence varies throughout the day and across seasons. Therefore, achieving stable solar spectral modulation over a wide range of incidence angles is crucial for the real-world implementation of such thin films. In this study, the average visible transmittance and average near-infrared reflectance of the films were assessed for both transverse electric (TE) and transverse magnetic (TM) polarized waves at various angles of incidence. This analysis enabled a comprehensive assessment of the robustness of the film's optical properties under varying illumination conditions.

### 2.5. Building Energy Consumption Simulation

To further evaluate the feasibility and energy-saving performance of the proposed film in practical applications, a simple office room model with dimensions of 5 m (length) × 4 m (width) × 3 m (height) was constructed using SketchUp (v2023, Trimble., Sunnyvale, CA, USA) [41], and its energy consumption was simulated using EnergyPlus [42]. The exterior walls of the building were composed of concrete blocks coated with cement plaster on both sides (see Table S2 for detailed parameters). Meteorological data for the simulations were obtained from the China Standard Weather Data (CSWD), corresponding to the climate zone of Changsha. As a city with a subtropical monsoon climate characterized by hot summers, Changsha provides a representative environment to assess the thermal regulation performance of the proposed film. The meteorological data were sourced from the official EnergyPlus website (v23.2.0, U.S. Department of Energy, Washington, DC, USA).

The internal load settings of the room are summarized in Table S3. In this study, a 5 mm-thick quartz glass window was selected as the reference glazing [43]. The annual energy consumption of the office was simulated under four window configurations: the reference window and windows coated with three, five, and seven layers of the THM film. Based on the simulation results, the energy saving rate (ESR) of the coated windows was calculated using Equation (7):

(7) $$ESR=\frac{Q_{\mathrm{ref}}-Q_{\mathrm{film}}}{Q_{\mathrm{ref}}}\times 100\%$$

where $Q_{\mathrm{ref}}$ represents the annual energy consumption of the office with the reference window, and $Q_{\mathrm{film}}$ denotes the annual energy consumption of the office with the window coated with the THM film.

## 3. Results and Discussion

### 3.1. Evaluation of the Optimized Film Performance

The multilayer film structures and their optical properties, obtained through inverse design using a DQN, are presented in Table S4. Among these, the Ta_2_O_5_/Ag/Ta_2_O_5_/Ag/Ta_2_O_5_ structure (42 nm/22 nm/79 nm/22 nm/40 nm) exhibits the highest SUM value, where $SUM={\bar{R}}_{\mathrm{UV}}+{\bar{T}}_{\mathrm{VIS}}+{\bar{R}}_{\mathrm{NIR}}$. Additionally, the structures with the highest SUM values in both the three-layer and seven-layer configurations also employed Ta_2_O_5_/Ag, indicating that the Ta_2_O_5_/Ag material combination consistently demonstrates superior performance in the design of THM films.

**Figure 3:**
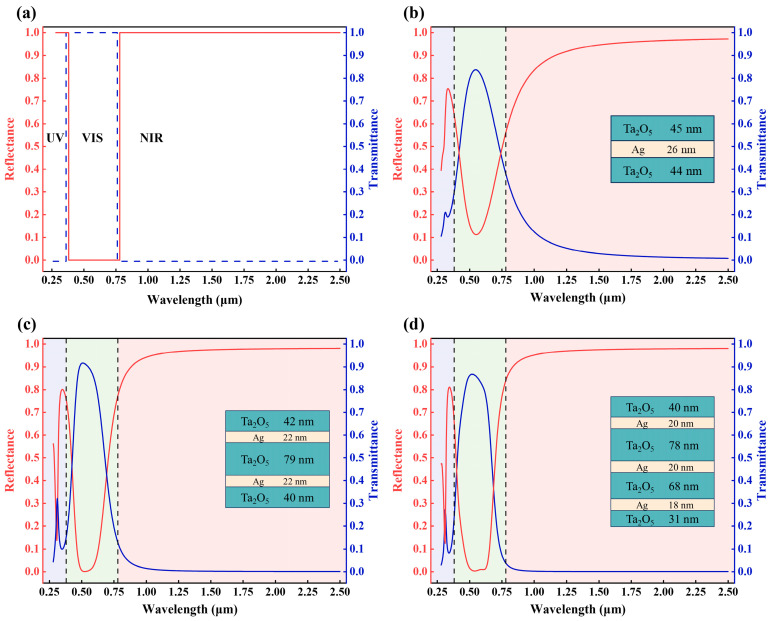
Reflectance and transmittance spectra of (**a**) ideal, (**b**) three-layer, (**c**) five-layer, and (**d**) seven-layer THM films over the wavelength range of 0.28–2.5 μm.

Figure 3a presents the reflectance and transmittance spectra of the ideal THM film. As shown in Figure 3c, the transmittance spectrum of the five-layer film peaks at 0.51 μm with a maximum transmittance of 92.0%. In contrast, the seven-layer film (Figure 3d) peaks at 0.52 μm, with a transmittance of 86.6%, which is 5.4% less than that of the five-layer film. Additionally, the transmittance of both films decreases to approximately 80.0% at 0.61 μm. According to Figure 1b, the human eye is particularly sensitive to visible light in the wavelength range of 0.51–0.61 μm. Consequently, the ${\bar{T}}_{\mathrm{VIS}}$ of the five-layer (87.0%) film is higher compared to that of the seven-layer film (83.3%). Notably, the reflectance spectrum of the seven-layer film exhibits a steeper rising slope, allowing for a faster transition from high visible transmittance to high near-infrared reflectance. At 0.78 μm, the reflectance of the seven-layer film increases to 84.0% compared to 77.0% for the five-layer film. Although their reflectance spectra nearly converge beyond a wavelength of 1 μm, the ${\bar{R}}_{\mathrm{NIR}}$ of the seven-layer film remains slightly higher (94.6%) than that of the five-layer film (93.2%). Furthermore, in the UV band, the five-layer film exhibits a broader high-reflectance bandwidth compared to the seven-layer film, leading to a relatively higher ${\bar{R}}_{\mathrm{UV}}$, which more effectively reduces UV transmittance and absorption. The three-layer film shown in Figure 3b demonstrates higher reflectance in the VIS range, resulting in a lower ${\bar{T}}_{\mathrm{VIS}}$. This is primarily due to the limited ability of the single-layer Ag film to reflect both UV and NIR radiation, leading to lower values of both ${\bar{R}}_{\mathrm{UV}}$ and ${\bar{R}}_{\mathrm{NIR}}$.

Therefore, the five-layer Ta_2_O_5_/Ag/Ta_2_O_5_/Ag/Ta_2_O_5_ structure demonstrates significantly better overall performance than the three-layer and seven-layer films, particularly in terms of spectral selectivity and modulation capability. As shown in Table 1, the THM film optimized via deep reinforcement learning achieves a more favorable balance between visible transmittance and NIR reflectance than those designed using conventional methods such as particle swarm optimization (PSO), resulting in superior overall spectral performance.

**Figure 4:**
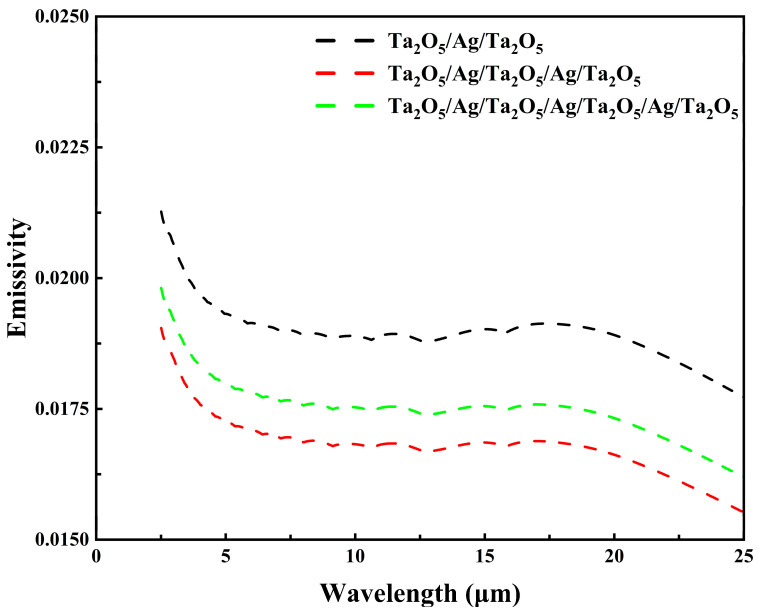
Emissivity spectra of the three-layer, five-layer, and seven-layer films in the mid- to far-infrared bands.

As shown in Figure 4, all three film structures exhibit relatively low emissivity in the mid- to far-infrared wavelength range (2.5–25 μm), with an overall decreasing trend as the wavelength increases. Among them, the five-layer structure shows the lowest emissivity, reaching as low as 0.01552 at a wavelength of 25 μm. Based on Equation (6), the average mid- to far-infrared emissivity values for the three-, five-, and seven-layer films are calculated to be 2.9%, 1.7%, and 2.1%, respectively, confirming that the five-layer film has the lowest average emissivity. This result can be attributed to the fact that the five-layer film exhibits the highest infrared reflectance, consequently achieving the lowest emissivity. This underscores its superior capability in suppressing long-wave thermal radiation compared to the other two designs.

### 3.2. Simulated Optical Properties

**Figure 5:**
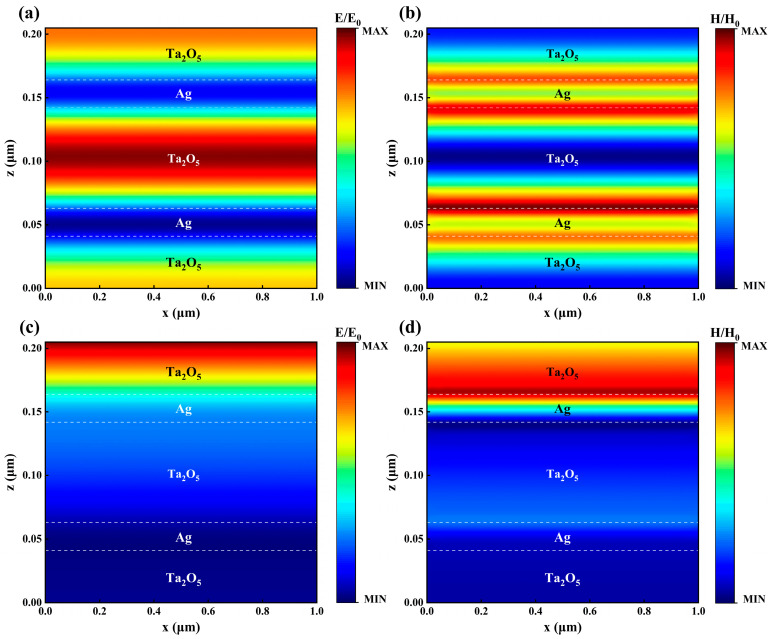
Electric and magnetic field distributions of the Ta_2_O_5_/Ag/Ta_2_O_5_/Ag/Ta_2_O_5_ structure. (**a**) Electric field distribution at a wavelength of 0.55 μm. (**b**) Magnetic field distribution at a wavelength of 0.55 μm. (**c**) Electric field distribution at a wavelength of 1 μm. (**d**) Magnetic field distribution at a wavelength of 1 μm.

The human eye is most sensitive to light at a wavelength of 0.55 μm. At this wavelength, the transmittance of the five-layer Ta_2_O_5_/Ag/Ta_2_O_5_/Ag/Ta_2_O_5_ film exceeds 90%, while the reflectance remains above 94% for wavelengths beyond 1 μm. To further investigate the underlying mechanisms, we analyzed the electric and magnetic field distributions at wavelengths of 0.55 μm and 1 μm. At a wavelength of 0.55 μm, the position where the electric field reaches its maximum intensity in Figure 5a corresponds to the location where the magnetic field exhibits its minimum intensity in Figure 5b. In addition, the electric and magnetic fields show opposite overall distribution trends. Although the upper silver layer reflects part of the incident visible light, a portion is transmitted into the Fabry–Pérot (F-P) cavity. Multiple internal reflections within the F-P cavity lead to the formation of standing wave modes. Destructive interference of the reflected waves at the interfaces suppresses reflection and consequently enhances the optical transmittance. This effect is known as the Fabry–Pérot resonance [45]. At a wavelength of 1 μm, the distribution trends of the electric field (Figure 5c) and magnetic field (Figure 5d) are approximately the same. This can be attributed to the large extinction coefficient (k) of Ag in the NIR band, which forms a highly reflective interface with the high-refractive-index Ta_2_O_5_ layer. As a result, nearly all the NIR light is reflected at the upper Ta_2_O_5_/Ag interface, causing some of the electric and magnetic field modes beneath the upper silver layer to converge toward a minimum.

**Figure 6:**
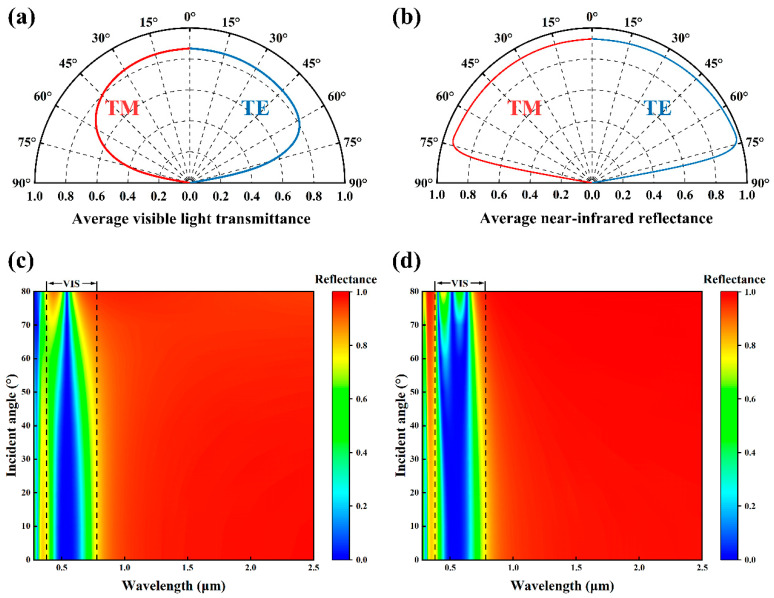
(**a**) Average visible light transmittance under the oblique incidence of TM and TE waves. (**b**) Average near-infrared reflectance under the oblique incidence of TM and TE waves. (**c**) Reflectance spectrum of TM waves under oblique incidence in the wavelength range of 0.28–2.5 μm. (**d**) Reflectance spectrum of TE waves under oblique incidence in the wavelength range of 0.28–2.5 μm.

According to Figure 6a,b, the trends of the ${\bar{T}}_{\mathrm{VIS}}$ and ${\bar{R}}_{\mathrm{NIR}}$ of the Ta_2_O_5_/Ag/Ta_2_O_5_/Ag/Ta_2_O_5_ thin film are generally similar for both TM and TE polarized waves at varying incident angles. When the incident angle is less than 60°, ${\overset{-}{T}}_{\mathrm{VIS}}$ consistently remains above 70%. Meanwhile, ${\bar{R}}_{\mathrm{NIR}}$ increases with the incident angle and reaches a maximum of 94% at 70°. However, when the incident angle exceeds 75°, both ${\bar{T}}_{\mathrm{VIS}}$ and ${\bar{R}}_{\mathrm{NIR}}$ drop sharply and approach zero at an incident angle of 90°. This indicates that the optical performance of the five-layer film is relatively insensitive to variations in the incident angle within a broad angular range (0–60°).

As shown in Figure 6c,d, in the VIS range, the low-reflectance bandwidth is relatively broad at normal incidence (0°), indicating a higher transmittance of visible light. As the angle of incidence increases, the low-reflectance bandwidth gradually narrows, i.e., the reflectance increases slightly. In the NIR band, however, the reflectance remains almost unaffected by variations in the angle of incidence. This optical behavior is of considerable significance in practical applications. At midday during summer, when sunlight strikes at a high angle, the increased reflectance of visible light helps reduce glare and thermal radiation, thereby improving indoor comfort. In contrast, during winter, when the solar incidence angle is low, the higher visible light transmittance facilitates both natural illumination and passive heat gain, contributing to a reduced heating demand.

### 3.3. Building Energy Performance

**Figure 7:**
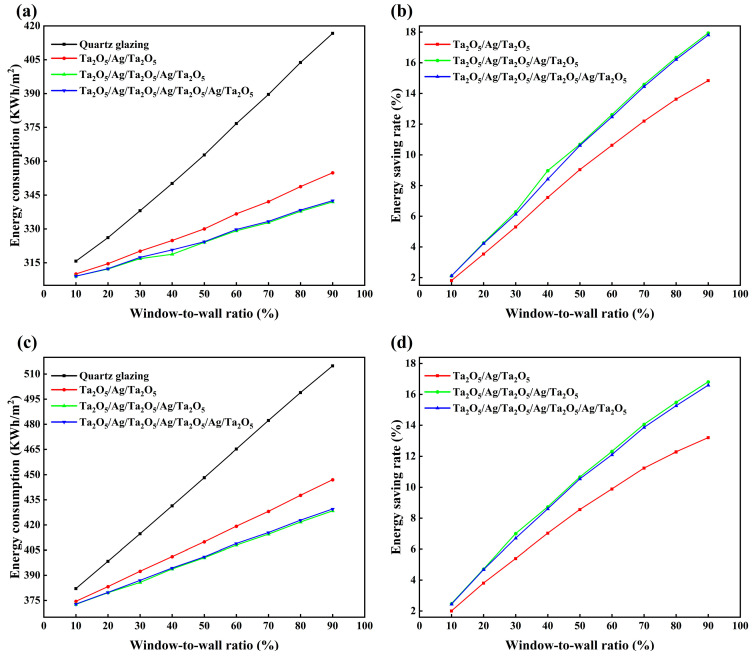
Under the climatic conditions of Changsha, (**a**) the annual building energy consumption per square meter as a function of window-to-wall ratio and (**b**) energy saving rate as a function of the window-to-wall ratio are shown. Under the climatic conditions of Guangzhou, (**c**) the annual building energy consumption per square meter as a function of window-to-wall ratio and (**d**) energy saving rate as a function of the window-to-wall ratio are shown.

Based on the variation in annual building energy consumption per square meter with the window-to-wall ratio shown in Figure 7a,c, it can be observed that windows coated with a five-layer Ta_2_O_5_/Ag/Ta_2_O_5_/Ag/Ta_2_O_5_ film exhibit the lowest energy consumption among the compared options. Meanwhile, as illustrated in Figure 7b,d, the energy efficiency of all three types of coated glass windows increases with the window-to-wall ratio. When the window-to-wall ratio reaches 90%, the five-layer coated windows achieve an energy savings rate of up to 17.93% under the climatic conditions of Changsha, which is 3.1% and 0.13% higher than the three-layer and seven-layer films, respectively. Under the climatic conditions of Guangzhou, the energy savings rate reaches 16.81%, exceeding those of the three-layer and seven-layer films by 3.61% and 0.22%, respectively. This result can be attributed to the superior optical performance of the five-layer structure. Its NIR is higher than that of the three-layer film, indicating a stronger capability to block infrared radiation and thereby reducing cooling energy consumption. Although the NIR of the seven-layer film is comparable to that of the five-layer structure, the latter exhibits a lower mid- to far-infrared emissivity. This characteristic helps suppress thermal radiation from the window into the indoor environment, effectively reducing heat transfer. Consequently, the five-layer configuration achieves the lowest energy consumption among the three designs, demonstrating the best overall energy-saving performance.

## 4. Conclusions

In this study, a film structure of Ta_2_O_5_/Ag/Ta_2_O_5_/Ag/Ta_2_O_5_ (42 nm/22 nm/79 nm/22 nm/40 nm) was inversely designed employing deep reinforcement learning to optimize both the material system and layer thickness parameters for broadband spectral selectivity. The film demonstrates high transmittance in the visible band (${\bar{T}}_{\mathrm{VIS}}$ = 87.0%) and high reflectance in the UV and NIR bands (${\bar{R}}_{\mathrm{UV}}$ = 75.5%, ${\bar{R}}_{\mathrm{NIR}}$ = 93.2%). Additionally, the film exhibits an average mid- to far-infrared emissivity as low as 1.7%, effectively reducing heat gain in summer and heat loss in winter. Simulation results under different polarization states and incidence angles indicate that the film maintains good optical performance across a wide angular range (0–60°). Under the hot climatic conditions of Changsha, China, windows coated with the proposed film outperform traditional quartz glass windows, achieving a maximum energy-saving rate of 17.93% at a window-to-wall ratio of 90%. Similarly, in Guangzhou, the energy-saving rate reaches 16.81%. The THM film developed in this study exhibits outstanding optical and thermal properties across the entire wavelength spectrum, indicating strong potential for application in energy-efficient building technologies.

## Figures and Tables

**Table 1:** Performance comparison between the THM design in this study and other related research.

Structure	${\bar{T}}_{\mathrm{VIS}}$ (%)	${\bar{R}}_{\mathrm{NIR}}$ (%)	Optimization Method	Reference
TiO_2_/Ag/TiO_2_	62.5	71.9	PSO	Dalapati et al. [19]
ZnO/Ag/ZnO	87.1	58.9	PSO	Dang et al. [33]
Si_3_N_4_/Ag/Si_3_N_4_	92.5	74.6	PSO	Hong et al. [14]
WO_3_/Au/WO_3_	79.0	60.3	PSO	Al-Kuhaili et al. [44]
Ta_2_O_5_/Ag/Ta_2_O_5_	83.0	81.7	DRL	This work
TiO_2_/Ag/TiO_2_/Ag/TiO_2_	87	93.2	DRL	This work

## Data Availability

The original contributions presented in this study are included in the supplementary material. Further inquiries can be directed to the corresponding author.

## Supplementary Materials

The following supporting information can be downloaded at: https://www.mdpi.com/article/10.3390/ma18122677/s1, Table S1: Type of material and its code [46,47,48,49,50,51,52,53,54,55,56,57,58], Table S2: Materials of the building façade, Table S3: Room internal load settings, Table S4: DQN-optimized THM film structure.
