# Supplementary material for: Deep Reinforcement Learning-Guided Inverse Design of Transparent Heat Mirror Film for Broadband Spectral Selectivity
Source: Materials (Basel). 2025 Jun 6;18(12):2677. doi: 10.3390/ma18122677 (PMC12194451; doi:10.3390/ma18122677)
Supplement: Supplementary file 1 [file materials-18-02677-s001.zip › materials-3631049-supplementary.pdf]

materials

Supplementary Materials

Deep Reinforcement Learning-Guided Inverse Design of Transparent Heat Mirror Film for Broadband Spectral Selectivity

Zhi Zeng, Haining Ji *, Tianjian Xiao, Peng Long, Bin Liu, Shisong Jin and Yuxin Cao

School of Physics and Optoelectronics, Xiangtan University, Xiangtan 411105, China; 202205710205@smail.xtu.edu.cn (Z.Z.); 202205710212@smail.xtu.edu.cn (T.X.); 202321521435@smail.xtu.edu.cn (P.L.); liubin@xtu.edu.cn (B.L.); 202421521418@smail.xtu.edu.cn (S.J.); 202421521397@smail.xtu.edu.cn (Y.C.)

* Correspondence: sdytjhn@xtu.edu.cn

The description of the mean squared error loss function

In the DQN model, we use the mean squared error (MSE) loss function to calculate the loss between the Q-value and the target Q-value. The formula is as follows:

$$\hat{Q} = r + \gamma(1 - done)\max Q_{\theta'}(s', a') \quad \text{(S1)}$$

In this equation (1), $\hat{Q}$ represents the target Q-value, r denotes the reward provided by the environment for the current state, and γ is the discount factor, which is set to 0.99 in this study. The variable $done$ is a Boolean indicating whether the episode has terminated. The termination condition is met when the time step reaches 2,000 or when the number of adjustments to the film structure exceeds 500 within an episode. The term $\max Q_{\theta'}(s', a')$ refers to the maximum Q-value in the next state, calculated by the target network.

Academic Editor: Dirk Poelman

Received: 22 April 2025
Revised: 2 June 2025
Accepted: 4 June 2025
Published: 6 June 2025

Citation: Zeng, Z.; Ji, H.; Xiao, T.; Long, P.; Liu, B.; Jin, S.; Cao, Y. Deep Reinforcement Learning-Guided Inverse Design of Transparent Heat Mirror Film for Broadband Spectral Selectivity. *Materials* **2025**, *18*, 2677. https://doi.org/10.3390/ma18122677

Copyright: © 2025 by the authors. Licensee MDPI, Basel, Switzerland. This article is an open access article distributed under the terms and conditions of the Creative Commons Attribution (CC BY) license (https://creativecommons.org/licenses/by/4.0/).

The description of the gradient descent algorithm

The weights of the main network are updated using the gradient descent algorithm. The corresponding formulas are as follows:

$$L = \frac{1}{N}\sum_{i=1}^{N}(\hat{Q}_i - Q_\theta(s_i, a_i))^2 \quad \text{(S2)}$$

$$\theta \leftarrow \theta_0 - \alpha\nabla_\theta L(\theta) \quad \text{(S3)}$$

In Equation (2), the batch is set to 64. $\hat{Q}_i$ denotes the target Q-value calculated according to Equation (1), and $Q_\theta(s_i, a_i)$ represents the current Q-value estimated by the main network for the state–action pair (s_i, a_i). In Equation (3), θ_0 refers to the weights of the main network before the update, and α is the learning rate. The initial value of α is set to 0.001 in this study, and it decays progressively as training proceeds. $\nabla_\theta L(\theta)$ denotes the gradient of the loss function with respect to the network parameters θ.

Detailed information on the candidate materials

To enable the model to distinguish between different materials, feature engineering was performed on the material names. Table S1 lists the integer code assigned to each material, along with the corresponding reference sources for the refractive index (n) and extinction coefficient (k) data.

Table S1. Type of material and its code.

Material Coding	Material Name	Reference	Material Coding	Material Name	Reference
0	Au	Ciesielski et al. [46]	8	Si_3N_4	Luke et al. [47]
1	Ag	Ciesielski et al. [46]	9	SiO_2	Gao et al. [48]
2	Cu	Babar et al. [49]	10	Ta_2O_5	Franta et al. [50]
3	TiN	Pflüger et al. [51]	11	TiO_2	Franta et al. [50]
4	Al_2O_3	Franta et al. [50]	12	ZnO	Aguilar et al. [52]
5	AlN	Yu et al. [53]	13	ZnS	Querry et al. [54]
6	HfO_2	Franta et al. [55]	14	WO_3	Kulikova et al. [56]
7	ITO	Minenkov et al. [57]	15	AZO	Treharne et al. [58]

Parameterization of building energy simulation

In this study, the exterior wall of the building is modeled as concrete coated with cement plaster on both sides. The specific material parameters are provided in Table S2.

Table S2. Materials of the building facade.

Parameter	Unit	Cement plaster	concrete block
Roughness	-	Smooth	Medium rough
Thickness	m	0.025	0.22
Conductivity	W (m·K)	0.730	0582
Density	Kg/m^3	1588	621
Specific heat	J/(Kg·K)	850	850
Thermal absorptance	-	0.9	0.9
Solar absorptance	-	0.4	0.5
Visible absorptance	-	0.4	0.4

Table S3. Room internal load settings.

Parameter	Unit	Value
Personnel density	people/m^2	0.54
Lighting power density	W/m^2	11.63
Power density of electrical equipment	W/m^2	48.01

Detailed information on the optimization results

Table S4. DQN-optimized THM film structure.

Structure	Thickness/nm	$\bar{R}_{UV}$	$\bar{T}_{VIS}$	$\bar{R}_{NIR}$	Sum
HfO_2/Ag/HfO_2	46/27/45	67.1%	79.7%	82.6%	2.294
Ta_2O_5/Ag/Ta_2O_5	45/26/44	70.8%	83.0%	81.7%	2.355
TiO_2/Ag/TiO_2/Ag/TiO_2	33/24/67/23/33	70.4%	88.5%	93.8%	2.527
Ta_2O_5/Ag/Ta_2O_5/Ag/Ta_2O_5	42/22/79/22/40	75.5%	87%	93.2%	2.557
HfO_2/Ag/HfO_2/Ag/HfO_2/Ag/HfO_2	150/10/64/20/80/20/31	73.3%	81.5%	93.3%	2.481
Ta_2O_5/Ag/Ta_2O_5/Ag/Ta_2O_5/Ag/Ta_2O_5	40/20/78/20/68/18/31	74.6%	83.3%	94.6%	2.525

References

46. Ciesielski, A.; Skowronski, L.; Trzcinski, M.; Górecka, E.; Trautman, P.; Szoplik, T. Evidence of germanium segregation in gold thin films. *Surface Science* **2018**, *674*, 73–78.
47. Luke, K.; Okawachi, Y.; Lamont, M. R.; Gaeta, A. L.; Lipson, M. Broadband mid-infrared frequency comb generation in a Si3N4 microresonator. *Optics letters* **2015**, *40* (21), 4823–4826.
48. Gao, L.; Lemarchand, F.; Lequime, M. Exploitation of multiple incidences spectrometric measurements for thin film reverse engineering. *Optics express* **2012**, *20* (14), 15734–15751.
49. Babar, S.; Weaver, J. Optical constants of Cu, Ag, and Au revisited. *Applied Optics* **2015**, *54* (3), 477–481.
50. Franta, D.; Nečas, D.; Ohlídal, I.; Giglia, A. Dispersion model for optical thin films applicable in wide spectral range. In *Optical Systems Design 2015: Optical Fabrication, Testing, and Metrology V*, **2015**, 9628, 342–353.
51. Pflüger, J.; Fink, J. Determination of optical constants by high-energy, electron-energy-loss spectroscopy (EELS). In *Handbook of Optical Constants of Solids*, Elsevier, **1997**, 293–311.
52. Aguilar, O.; de Castro, S.; Godoy, M. P.; Rebello Sousa Dias, M. Optoelectronic characterization of Zn1-xCdxO thin films as an alternative to photonic crystals in organic solar cells. *Optical Materials Express* **2019**, *9* (9), 3638–3648.
53. Beliaev, L. Y.; Shkondin, E.; Lavrinenko, A. V.; Takayama, O. Thickness-dependent optical properties of aluminum nitride films for mid-infrared wavelengths. *Journal of Vacuum Science & Technology A* **2021**, *39* (4), 043408.
54. Ozaki, S. O. S.; Adachi, S. A. S. Optical constants of cubic ZnS. *Japanese journal of applied physics* **1993**, *32* (11R), 5008.
55. Franta, D.; Nečas, D.; Ohlídal, I. Universal dispersion model for characterization of optical thin films over a wide spectral range: application to hafnia. *Applied Optics* **2015**, *54* (31), 9108–9119.
56. Kulikova, D. P.; Dobronosova, A. A.; Kornienko, V. V.; Nechepurenko, I. A.; Baburin, A. S.; Sergeev, E. V.; Lotkov, E. S.; Rodionov, I. A.; Baryshev, A. V.; Dorofeenko, A. V. Optical properties of tungsten trioxide, palladium, and platinum thin films for functional nanostructures engineering. *Optics express* **2020**, *28* (21), 32049–32060.
57. Minenkov, A.; Hollweger, S.; Duchoslav, J.; Erdene-Ochir, O.; Weise, M.; Ermilova, E.; Hertwig, A.; Schiek, M. Monitoring the electrochemical failure of indium tin oxide electrodes via operando ellipsometry complemented by electron microscopy and spectroscopy. *ACS Applied Materials & Interfaces* **2024**, *16* (7), 9517–9531.
58. Treharne, R.; Seymour-Pierce, A.; Durose, K.; Hutchings, K.; Roncallo, S.; Lane, D. Optical design and fabrication of fully sputtered CdTe/CdS solar cells. In *Journal of Physics: Conference Series*, **2011**, 286, 012038.
